# Hydroxychloroquine plus standard of care compared with standard of care alone in COVID-19: a meta-analysis of randomized controlled trials

**DOI:** 10.1038/s41598-021-91089-3

**Published:** 2021-06-07

**Authors:** Bahman Amani, Ahmad Khanijahani, Behnam Amani

**Affiliations:** 1 Health Management and Economics Research Center, Health Management Research Institute, Iran University of Medical Sciences, Tehran, Iran; 2 John G. Rangos School of Health Sciences, Duquesne University, 600 Forbes Ave, Pittsburgh, PA 15282 USA

**Keywords:** Medical research, Outcomes research

## Abstract

The efficacy and safety of Hydroxychloroquine (HCQ) in treating coronavirus disease (COVID-19) is disputed. This systematic review and meta-analysis aimed to examine the efficacy and safety of HCQ in addition to standard of care (SOC) in COVID-19. PubMed, the Cochrane Library, Embase, Web of sciences, and medRxiv were searched up to March 15, 2021. Clinical studies registry databases were also searched for identifying potential clinical trials. The references list of the key studies was reviewed to identify additional relevant resources. The quality of the included studies was evaluated using the Cochrane Collaboration tool and Jadad checklist. Meta-analysis was performed using RevMan software (version 5.3). Eleven randomized controlled trials with a total number of 8161 patients were identified as eligible for meta-analysis. No significant differences were observed between the two treatment groups in terms of negative rate of polymerase chain reaction (PCR) (Risk ratio [RR]: 0.99, 95% confidence interval (CI) 0.90, 1.08; P = 0.76), PCR negative conversion time (Mean difference [MD]: − 1.06, 95% CI − 3.10, 0.97; P = 0.30), all-cause mortality (RR: 1.09, 95% CI 1.00, 1.20; P = 0.06), body temperature recovery time (MD: − 0.64, 95% CI − 1.37, 0.10; P = 0.09), length of hospital stay (MD: − 0.17, 95% CI − 0.80, 0.46; P = 0.59), use of mechanical ventilation (RR: 1.12, 95% CI 0.95, 1.32; P = 0.19), and disease progression (RR = 0.82, 95% CI 0.37, 1.85; P = 0.64). However, there was a significant difference between two groups regarding adverse events (RR: 1.81, 95% CI 1.36, 2.42; P < 0.05). The findings suggest that the addition of HCQ to SOC has no benefit in the treatment of hospitalized patients with COVID-19. Additionally, it is associated with more adverse events.

## Introduction

The COVID-19 pandemic was identified and reported for the first time in Wuhan, China^1–3^, and has been recognized as a global public health concern by the World Health Organization (WHO)^4^. The mortality of critically ill patients with COVID-19 is considerable^5^. The initial estimations for the case fatality rate were about 2.3% in China^6^ and 7.2% in Italy^7^. The 2019-nCoV infection causes clusters of severe respiratory illnesses similar to severe acute respiratory syndrome (SARS) coronavirus and, in severe cases, is associated with hospitalization, ICU admission, and frequent mortalities^8, 9^. Fever, coughing, shortness of breath, muscle ache, confusion, headache, sore throat, rhinorrhea, chest pain, diarrhea, nausea, and vomiting are among the clinical manifestations of the disease^10^. Early efforts have focused on describing the clinical course, containing severe cases, and treating the sick^11^.

There are several options for controlling and preventing the development of COVID-19 infections, including vaccines, monoclonal antibodies, oligonucleotide-based therapies, peptides, interferon therapies, and small-molecule drugs^5^. Lopinavir/Ritonavir (400/100 mg every 12 h), Chloroquine (500 mg every 12 h), and Hydroxychloroquine (HCQ) (200 mg every 12 h) and Alpha-interferon are also proposed as courses of treatment^12^.

HCQ is used for a variety of diseases, including IgAN^13, 14^, Arthritis^15, 16^, Pulmonary Sarcoidosis^17^, Cutaneous lupus erythematosus^18–20^, Sjogren’s syndrome^21^, and Type 2 diabetes mellitus^22^. HCQ prevents the activity of lysosomal enzymes. This drug can reduce the production of cytokines, including type 1 interferons, and inhibit T cell activation and the differentiation and expression of excitatory molecules (CD154)^23^. Currently, several observational^24, 25^ and clinical studies^26, 27^ have evaluated the efficacy of HCQ on COVID-19. Besides, several meta-analyses examined HCQ in COVID-19 regarding a few of the outcomes, such as mortality and negative rate of polymerase chain reaction (PCR). Therefore, there is an urgent need to examine more detailed outcomes based on the available evidence. The purpose of this study was to examine whether HCQ in addition to standard of care (SOC) versus SOC alone is more effective and safer in hospitalized patients with COVID-19.

## Methods

The study protocol was registered in the International Prospective Register of Systematic Reviews (PROSPERO) with the registration number CRD42020179425. When writing this report, we used the preferred reporting items for systematic reviews and meta-analysis (PRISMA)^28^. The PRISMA statement consists of a 27-item checklist and a 4-phase flow diagram. We used the PRISMA checklist to prepare the report and diagram for the screening process.

### Search strategy

A systematic review of the relevant literature was conducted in PubMed, The Cochrane Library, Embase, Web of Sciences, and medRxiv up to March 15, 2021. In order to increase the sensitivity of the search, Google Scholar was also searched. In addition, the European Union Clinical Trials Register, Clinical trial Gov. and Chinese Clinical Trial Registry (ChiCTR) were searched. Finally, the references list of the final studies and review articles were reviewed for more citations. The search terms used were 2019-nCoV, SARS-CoV-2, COVID-19, and Hydroxychloroquine, which were usually limited to the title and the abstract of the articles. We included articles with English full-text or abstract.

The following is our search strategy used to search for relevant articles published in PubMed: ((((((COVID-19[Title/Abstract]) OR (SARS-CoV-2[Title/Abstract])) OR (Coronavirus[Title/Abstract])) OR (Coronavirus[MeSH Terms])) OR (2019-nCoV[Title/Abstract])) OR (Novel coronavirus[Title/Abstract])) AND (Hydroxychloroquine[Title/Abstract]). We followed a similar logic while performing searches in other databases.

### Study selection

Two researchers independently screened the identified studies based on the inclusion criteria. Disagreements were resolved by discussion among the authors. The following inclusion criteria were used for selecting the articles: (1) hospitalized patients with suspected or confirmed COVID-19 by laboratory tests; (2) HCQ plus SOC as intervention; (3) SOC alone as control; (4) randomized controlled trial as study design; (5) published as abstract or full-text; and (6) primary and secondary outcomes of interests (negative conversion time, negative rate of PCR, mortality rate, body temperature recovery time, length of hospital stay, and any adverse events). Other studies and reports such as letters to the editor, case reports, editorial comments, observational studies, and animal models were excluded.

### Data extraction and quality assessment

The Cochrane risk of bias tool was used to assess the risk of bias in five domains (selection, performance, attrition, reporting, and other) in the included studies. We used the Jadad checklist to evaluate the quality of clinical trial studies. Information on the study characteristics (place, design, and duration); patient’s characteristics (age, sex, and the number of patients); intervention and control (treatment protocol); efficacy outcomes; and adverse events were extracted. Both processes were independently performed by two authors, and disagreements were resolved by discussion among the authors.

### Quantitative data synthesis

A meta-analysis was conducted to compare the efficacy and safety of HCQ plus SOC versus SOC alone using the RevMan (version 5.3) software which is recommended by the Cochrane Handbook for Systematic Reviews of Interventions. The mean difference (MD) and risk ratio (RR) with a 95% confidence interval (CI) were used for continuous and dichotomous variables, respectively. Statistical heterogeneity was evaluated using I-square > 50% and Chi-square with a significance level p < 0.1. The random-effects method was used for statistical heterogeneity; otherwise, the fixed-effect method was used.

## Results

### Study characteristics

**Figure 1:**
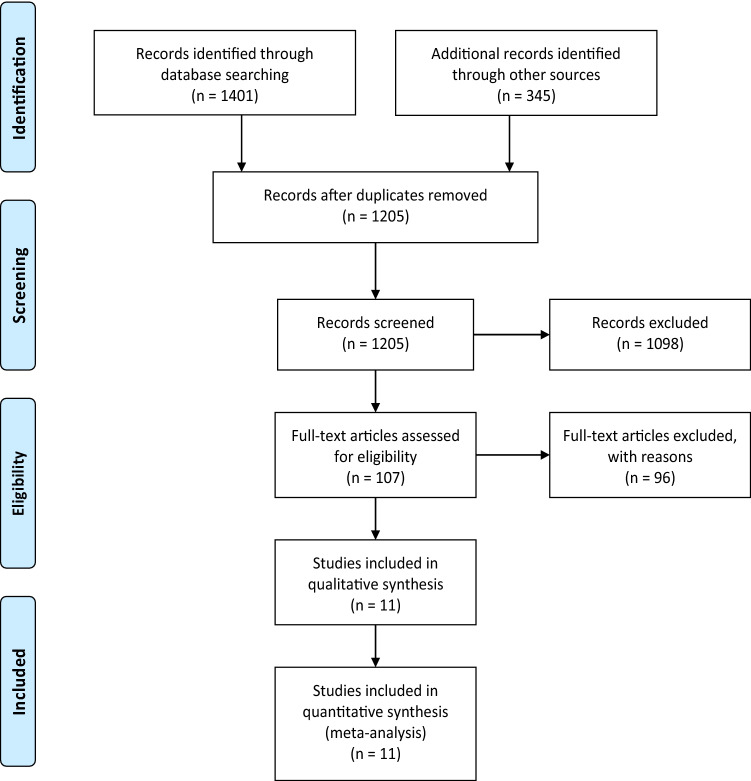
Flowchart of the study selection process.

**Figure 2:**
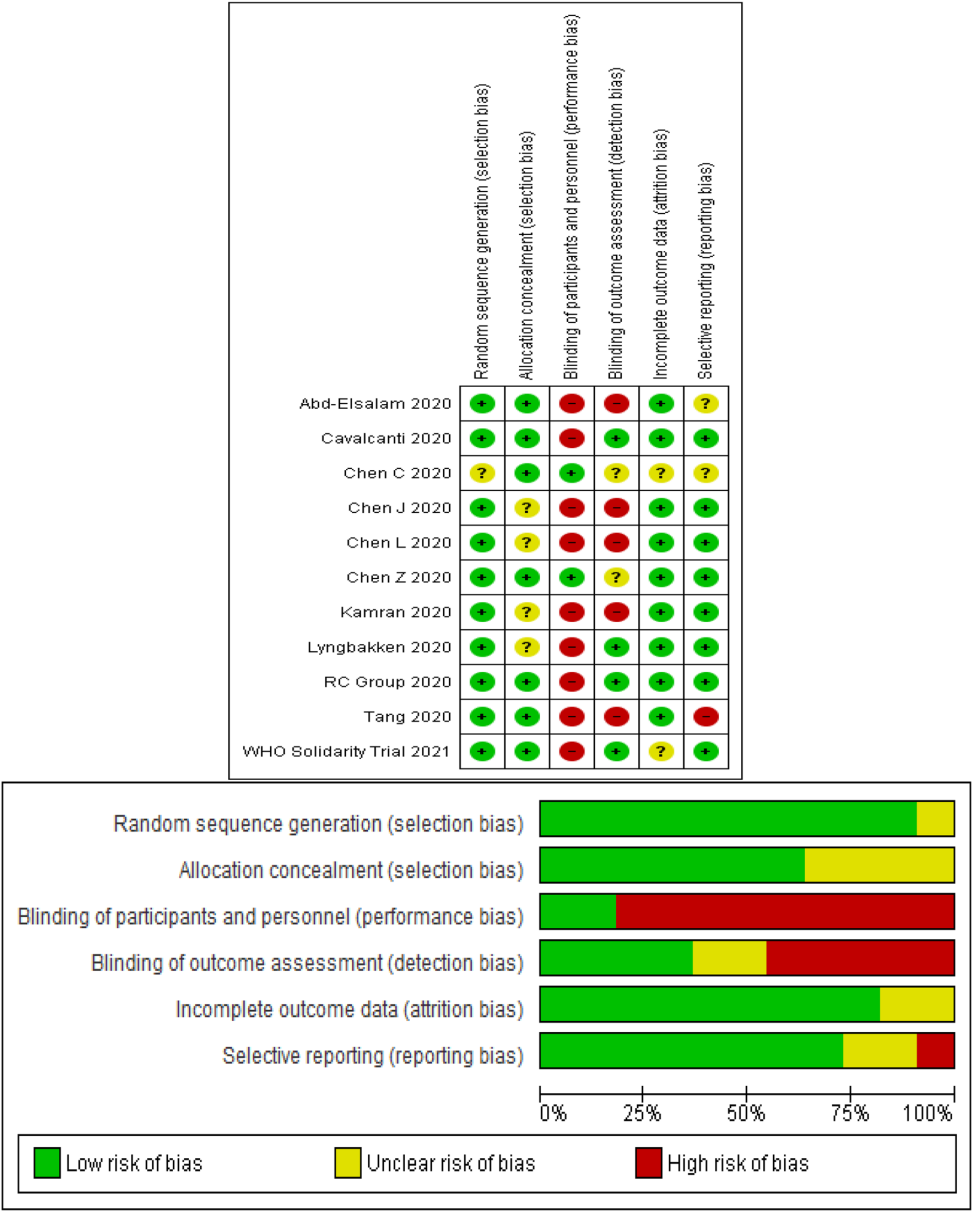
Risk of bias in the selected studies.

Figure 1 shows the process of literature search, removal of duplication, and screening. Of the total 1,205 records, 1,098 were excluded based on the title and abstract. The remaining 107 records were review for full text. Finally, eleven randomized controlled trials (RCTs)^26, 29–38^ with 8161 patients were included in the meta-analysis. The main characteristics of the selected studies are presented in Table 1. Assessment of the risk of bias using the Cochrane Collaboration tool is presented in Fig. 2.

**Table 1:** The characteristics of RCTs included in the meta-analysis.

Author, year	Place	Patients (N, male)	Age	Diagnosis	Intervention (N, HCQ protocol)	SOC (N, protocol)	Jadad scale
Abd-Elsalam, 2020	Egypt	194,118	40.72	PCR	97; 400 mg twice/day (in day 1) followed by 200 mg tablets twice/day + SOC	97; Paracetamol, oxygen, fluids, empiric antibiotic, oseltamivir, and invasive mechanical ventilation	3
Chen Z, 2020	China	62, 29	44.7	RT-PCR	31; (400 mg/day (200 mg/bid) between days 1 and 5) + SOC	31; Oxygen therapy, antiviral and antibacterial agents, immunoglobulin, corticosteroids	2
Chen J, 2020	China	30, 21	48.6	PCR	15; (400 mg per day for 5 days) + SOC	15; O2 therapy, interferon, lopinavir/ ritonavir, antibiotics, and supportive treatment	2
Chen CP, 2020	Taiwan	33, 19	32.9	RT-PCR	21; HCQ 400 mg twice for 1 day and HCQ 200 mg twice daily for 6 days + SOC	12; Ceftriaxone, azithromycin, levofloxacin, moxifloxacin, Oseltamivir	2
Chen L, 2020	China	66, 15	NR	RT-PCR or CT	18; 200 mg Bid for 10 days	12; NR	3
Cavalcanti, 2020	Brazil	504, 265	50.3	NR	221; HCQ 400 mg twice daily, for 7 days + SOC	227; Glucocorticoids, immunomodulators, antibiotic and antiviral agents	4
Kamran, 2020	Pakistan	500,466	35.96	PCR	349; HCQ 400 mg twice a day for day one followed by 200 mg 12 hourly for next 5 days + SOC	151; Vit C, zinc, Vit-D, Paracetamol, intravenous fluids, hemodynamic monitoring parameters	3
Lyngbakken, 2020	Norway	53, 35	62	RT-PCR	27; HCQ 400 mg twice for 7 days + SOC	26; NR	2
RC Group,2020	UK	4716, 2935	NR	NR	1561; 800 mg at 0 and 6 h; 400 mg at 12 h; 400 mg × 9 days + SOC	3155; NR	2
Tang, 2020	China	150, 82	46	Real time RT-PCR	75; HCQ 1200 mg/day × 3 day followed by 800/day + SOC	75; Intravenous fluids, supplemental oxygen, , hemodynamic monitoring, and intensive care	3
WHO Solidarity Trial, 2021	30 Countries	1853,1109	NR	NR	947,800 mg at 0 and hour 6, starting at hour 12, 800 mg for 10 days	906; NR	2

CT: Computed tomography, HCQ: Hydroxychloroquine, NR: Not reported, PCR: Polymerase chain reaction, RT-PCR: Reverse transcription polymerase chain reaction.

### Outcomes

#### Primary outcomes

**Figure 3:**
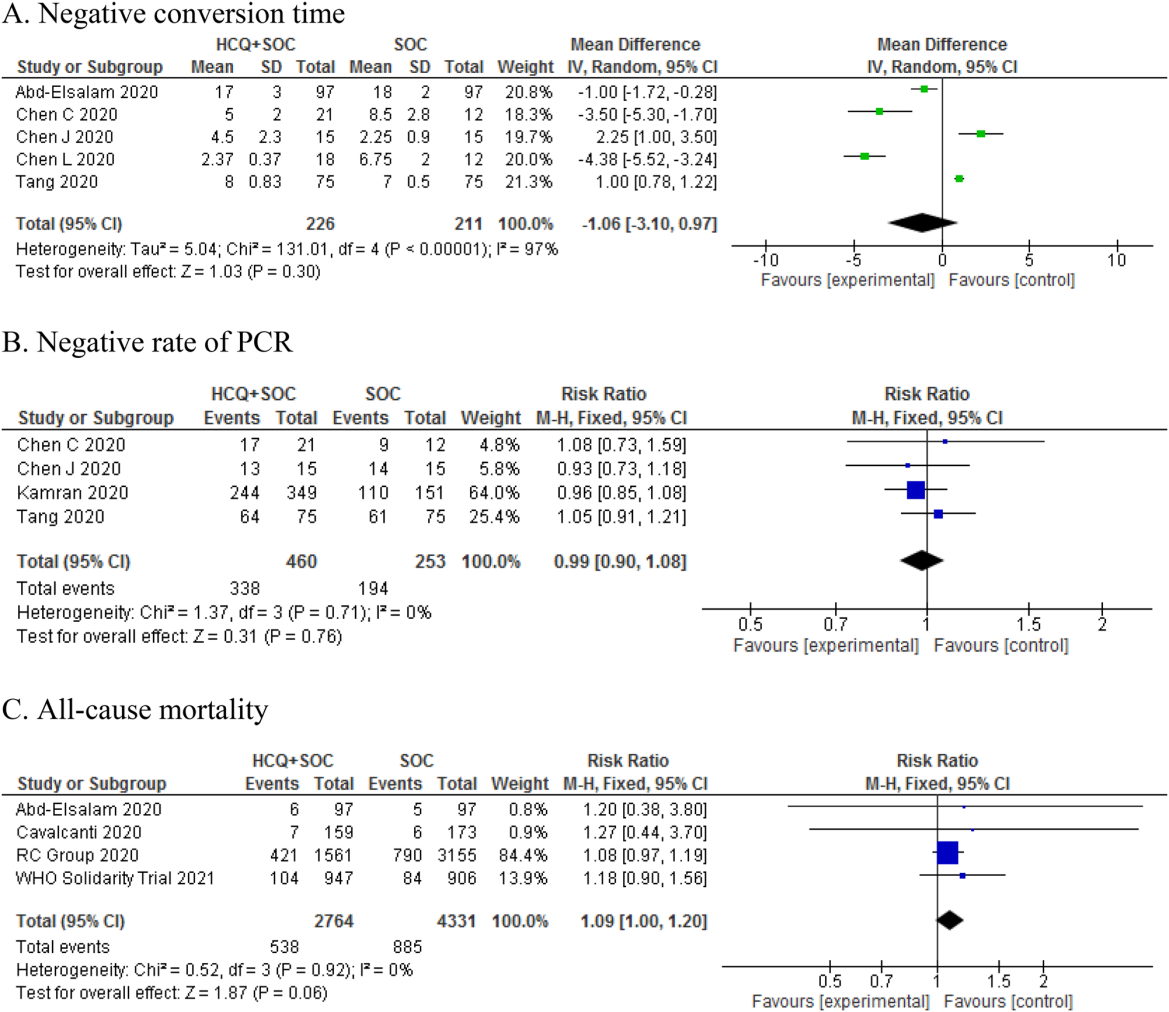
Forest plot of HCQ plus SOC vs. SOC for primary outcomes; Negative conversion time (**A**), Negative rate of PCR (**B**), and All-cause mortality (**C**) in COVID-19 patients.

The meta-analysis demonstrated that there was no significant difference between the HCQ plus SOC group and SOC group in terms of the negative rate of PCR (RR: 0.99, 95% CI 0.90, 1.08; P = 0.76), PCR negative conversion time (MD: − 1.06, 95% CI − 3.10, 0.97; P = 0.30), and all-cause mortality (RR: 1.13, 95% CI 0.99 1.27, P = 0.06) (Fig. 3).

#### Secondary outcomes

**Figure 4:**
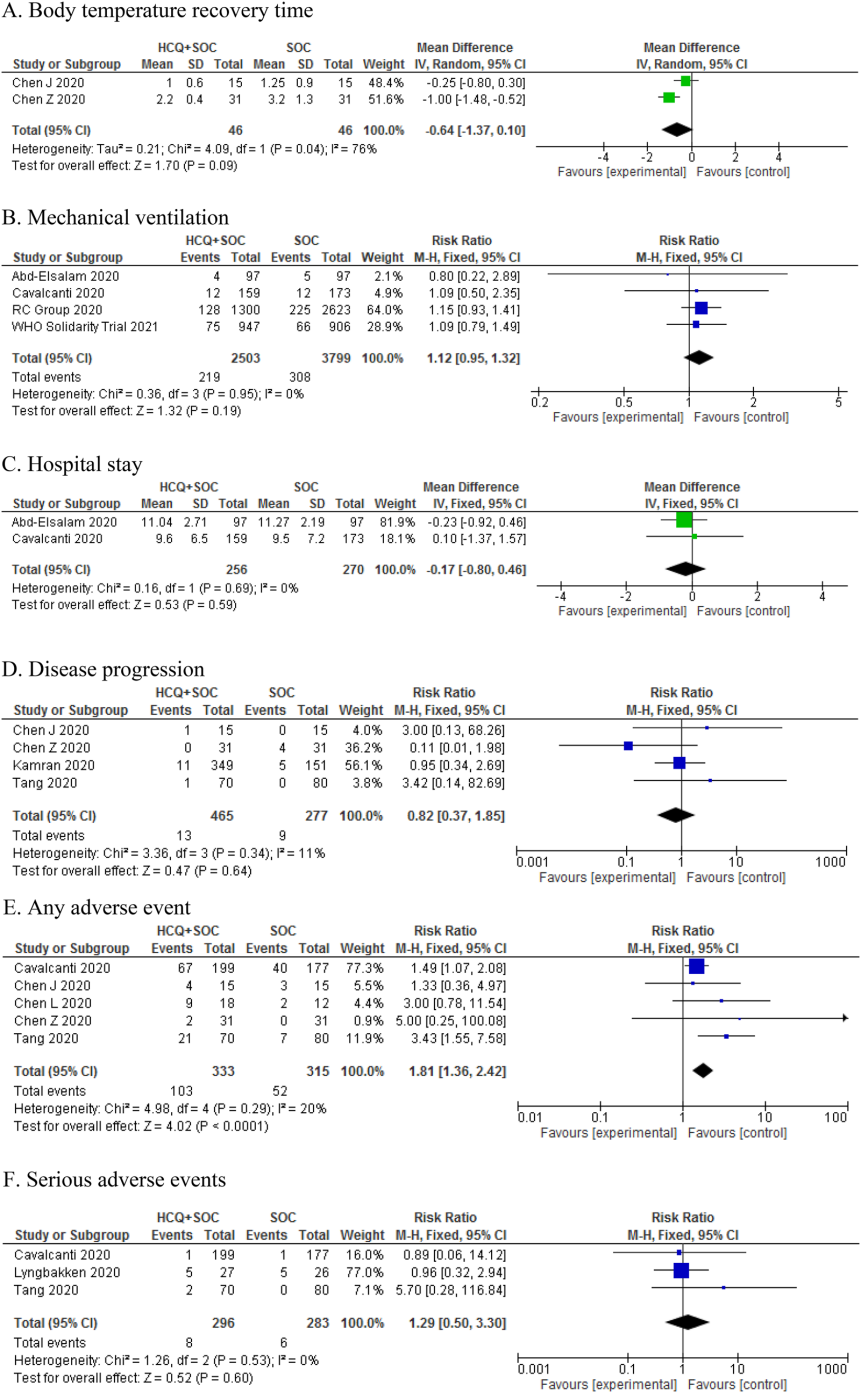
Forest plot of HCQ plus SOC vs. SOC for primary outcomes; Body temperature recovery time (**A**), Mechanical ventilation (**B**), Hospital stay (**C**), Disease progression (**D**), Any adverse event (**E**), and Serious adverse events (**F**) in COVID-19 patients.

The pooled RR of included studies showed that adding HCQ to SOC was not associated with significant effect on secondary outcomes including body temperature recovery time (MD: − 0.64, 95% CI − 1.37, 0.10; P = 0.09), the length of hospital stay (MD: − 0.17, 95% CI − 0.80 0.46, P = 0.59), the use of mechanical ventilation (RR: 1.12, 95% CI 0.95, 1.32; P = 0.19), and disease progression (RR: 0.82, 95% CI 0.37, 1.85; P = 0.64) (Fig. 3). The pooled RR of 5 studies showed that the addition of HCQ to SOC was associated with higher rates of adverse events in hospitalized patients (RR: 1.81, 95% CI 1.36, 2.42; P < 0.05) (Fig. 4). However, there was no significant difference between the two groups in terms of serious adverse events (RR: 1.29, 95% CI 0.50, 3.30; P = 0.60) (Fig. 4).

### Sensitivity analysis and subgroup analysis

The findings of the subgroup analysis are presented in Table 2. The findings showed that primary outcomes did not change regarding dose, sample size, treatment duration, and severity of COVID-19. We performed a sensitivity analysis based on different settings and control groups. For this reason, we included RCTs done on non-hospitalized patients and RCTs with placebo as control. The result did not change in terms of the negative rate of PCR and mortality rate (Table 2).

**Table 2:** Sensitivity analysis and subgroup analysis for primary outcomes.

Analysis	No. of studies	MD or RR (95% CI)	P	Heterogeneity		
				Chi^2^	P	I^2^
**Sensitivity analysis**						
Negative rate of PCR	5	0.96 [0.87, 1.06]	0.45	0.93	0.92	0%
Mortality	9	1.07 [0.98, 1.17]	0.13	3.45	0.90	0%
**Subgroup analysis**						
PCR Negative conversion time						
Dose	4					
400 mg/day^a^	2	− 1.07 [− 7.57, 5.43]	0.75	58.80	< 0.05	98%
> 400 mg/day on first day^b^	2	− 2.11 [− 4.54, 0.33]	0.09	6.39	0.01	84%
**Treatment duration with HCQ**	5					
< 7 days	2	0.23 [− 1.37, 1.82]	0.84	26.44	< 0.05	96%
≥ 7 days	3	− 1.41 [− 4.08, 1.27]	0.34	104.30	< 0.05	98%
**Sample size**	5					
< 100	3	− 1.87 [− 6.30, 2.57]	0.41	63.26	< 0.05	97%
≥ 100	2	0.03 [− 1.93, 1.99]	0.98	27.30	< 0.05	96%
**Severe of COVID-19**	5					
Mild/moderate	3	0.07 [− 2.21, 2.34]	0.96	27.87	< 0.05	93%
Moderate	1	− 4.38 [− 5.52, − 3.24]	< 0.05	–	–	–
Mild/moderate/severe	1	− 1.00 [− 1.72, − 0.28]	< 0.05	–	–	–
**Negative rate of PCR**						
At two time points	4					
On day 7	3	1.02 [0.69, 1.52]	0.91	13.73	0.001	85%
On day 14	3	0.97 [0.89, 1.07]	0.58	0.35	0.84	0%
**Sample size**	4					
< 100	2	1.00 [0.80, 1.24]	0.97	0.50	0.48	0%
≥ 100	2	0.97 [0.87, 1.08]	0.58	0.08	0.92	0%
**Severe of COVID-19**	4					
Mild/moderate	3	1.00 [0.80, 1.24]	0.97	0.50	0.48	0%
Mild	1	0.96 [0.85, 1.08]	0.05	–	–	–

CI: confidence interval, HCQ: hydroxychloroquine, MD: mean difference, PCR: polymerase chain reaction, RR: risk ratio.

^a^Patients were given a fixed-dose 400 mg/day during treatment.

^b^Patients were given a dose higher than 400 mg on the first day, then a fixed dose of 400 mg/day for other days.

## Discussion

The purpose of this study was to examine the efficacy and safety of HCQ plus SOC compared to SOC alone for COVID-19.

Several RCTs have examined the efficacy and safety of HCQ in treating COVID-19. The findings of our meta-analysis showed that the addition of HCQ to SOC in patients with COVID-19 was not associated with significant improvement in any outcomes reported in patients. Previously published meta-analyses^39–42^ on observational studies and RCTs also found no clinical benefits for HCQ in comparison with SOC for COVID-19 patients, which confirms our findings. Gautret et al.^43^ suggested that the causes of insufficient response to treatment with HCQ in the non-respondents with COVID-19 should be examined by factors such as SARS-CoV-2 strains, genome, and other associated factors with the metabolism of HCQ in patients. A possible mechanism for HCQ inefficiency was explained by Sandeep and McGregor^44^ using virtualized quantum mechanical modeling. However, Yao et al.^45^ found that HCQ was more potent than Chloroquine in inhabiting SARS-CoV-2 in vitro.

It should be noted that most of the included studies in these meta-analyses were observational. There are some concerns regarding the limitations of these studies that should be considered. All kinds of biases such as confounding, reverse causation, statistical considerations, and other issues are limitations of these studies in the estimation of drug efficacy and safety^46^. The Agency for Healthcare Research and Quality (AHRQ) has provided recommendations on including observational studies in the comparative effectiveness review process for comparing medical interventions^30^. Our meta-analysis showed that HCQ was not associated with a significant reduction or increase in the COVID-19 mortality rate. The results from similar meta-analyses^40, 47^ are in line with our findings. Moreover, we conducted a sensitivity analysis of studies done in nonhospital patients and placebo as a control, plus included RCTs regarding the negative rate of PCR and mortality rate. The result did not show any significant differences. Fiolet et al.^47^ also found that HCQ was not associated with a different mortality rate in all studies and RCTs, which confirms our findings.

Some studies^43, 48^ have supported the synergistic effect of HCQ with Azithromycin on COVID-19. In an open-label non-randomized clinical trial, Gautret et al.^43^ found that 100% of patients who received HCQ and Azithromycin as combination therapy successfully recovered from COVID-19. These authors found similar results in another study. A meta-analysis showed that HCQ alone or in combination with Azithromycin in comparison with the control group was not effective in treating COVID-19 and was associated with higher mortality rates. It seems that there is no benefit for HCQ in combination with Azithromycin, and it is associated with an increased mortality rate^47^. Contradictorily, a study on 1,438 patients hospitalized with Covid-19 in all United States Veterans Health Administration medical centers found that the rates of death in patients under treatment with HCQ alone were higher than and HCQ plus Azithromycin (27.8%, 22.1%)^25^. However, a meta-analysis of the adverse effects of long-term Azithromycin use in patients with chronic lung diseases showed that Azithromycin is associated with an increased risk of bacterial resistance (2.7-fold)^49^.

Our meta-analysis showed that as an add-on therapy to SOC was associated with more adverse events. Reported adverse events in RCTs were mainly mild. Three RCTs reported serious adverse events in patients taking HCQ and SOC. However, this difference was not *statistically* significant. Generally, adverse effects of antimalarial are usually rare and mild^50^. Gastric symptoms are a prevalent adverse effect of HCQ^51, 52^; other adverse events include Cutaneous^53^, headache^54–56^, Cardiomyopathy^57–59^, and Retinopathy^60–64^. Regarding the short-term follow-up of the studies, it is recommended that patients who received HCQ should be monitored for possible adverse events over a longer period of time.

### Limitations

High heterogeneity between studies, the use of different treatment protocols as SOC, short follow-up periods, and lack of rigorous methodologies of the studies were among the limitations of our study. Nevertheless, the findings of this study can be beneficial for guiding clinicians in decisions regarding COVID-19 treatment.

## Conclusion

According to the findings of this study, HCQ in addition to SOC appears to be not an effective treatment in primary outcomes, including negative conversion time, the negative rate of PCR, and mortality rate. In addition, HCQ did not show a significant improvement in other secondary outcomes. HCQ was also associated with higher rates of adverse events.

## References

[1] Nishiga M, Wang DW, Han Y, Lewis DB, Wu JC (2020). COVID-19 and cardiovascular disease: From basic mechanisms to clinical perspectives. Nat. Rev. Cardiol..

[2] Ayres JS (2020). A metabolic handbook for the COVID-19 pandemic. Nat. Metab..

[3] Rao, K. *et al.* Review on newly identified coronavirus and its genomic organization. (2020).

[4] de Souza WM (2020). Epidemiological and clinical characteristics of the COVID-19 epidemic in Brazil. Nat. Hum. Behav..

[5] Yang X (2020). Clinical course and outcomes of critically ill patients with SARS-CoV-2 pneumonia in Wuhan, China: A single-centered, retrospective, observational study. Lancet Respir. Med..

[6] Wu Z, McGoogan JM (2020). Characteristics of and important lessons from the coronavirus disease 2019 (COVID-19) outbreak in China: Summary of a report of 72 314 cases from the Chinese Center for Disease Control and Prevention. JAMA.

[7] Livingston E, Bucher K (2020). Coronavirus disease 2019 (COVID-19) in Italy. JAMA.

[8] Huang C (2020). Clinical features of patients infected with 2019 novel coronavirus in Wuhan, China. Lancet.

[9] COVID, C, Team, R (2020). Severe outcomes among patients with coronavirus disease 2019 (COVID-19)—United States, February 12–March 16, 2020. MMWR Morb. Mortal Wkly. Rep..

[10] Chen N (2020). Epidemiological and clinical characteristics of 99 cases of 2019 novel coronavirus pneumonia in Wuhan, China: A descriptive study. Lancet.

[11] Lipsitch M, Swerdlow DL, Finelli L (2020). Defining the epidemiology of Covid-19—studies needed. N. Engl. J. Med..

[12] Cascella, M., Rajnik, M., Cuomo, A., Dulebohn, S. C. & Di Napoli, R. In *Statpearls [internet]* (StatPearls Publishing, 2020).

[13] Yang Y-Z (2018). Effects of hydroxychloroquine on proteinuria in immunoglobulin A nephropathy. Am. J. Nephrol..

[14] Liu L-J (2019). Effects of hydroxychloroquine on proteinuria in IgA nephropathy: A randomized controlled trial. Am. J. Kidney Dis..

[15] Ravindran V, Alias G (2017). Efficacy of combination DMARD therapy vs hydroxychloroquine monotherapy in chronic persistent chikungunya arthritis: A 24-week randomized controlled open label study. Clin. Rheumatol..

[16] Schapink L, van den Ende CH, Gevers LA, van Ede AE, den Broeder AA (2019). The effects of methotrexate and hydroxychloroquine combination therapy vs methotrexate monotherapy in early rheumatoid arthritis patients. Rheumatology.

[17] McDonnell, M., Suleem, I., Rutherford, R., O’Regan, A. & Gilmartin, J. (Eur Respiratory Soc, 2011).

[18] Yokogawa N (2017). Effects of hydroxychloroquine in patients with cutaneous lupus erythematosus: A multicenter, double-blind, randomized, parallel-group trial. Arthritis Rheumatol..

[19] Ikeda T, Kanazawa N, Furukawa F (2012). Hydroxychloroquine administration for Japanese lupus erythematosus in Wakayama: A pilot study. J. Dermatol..

[20] Yokogawa N (2013). Response to hydroxychloroquine in Japanese patients with lupus-related skin disease using the cutaneous lupus erythematosus disease area and severity index (CLASI). Mod. Rheumatol..

[21] van der Heijden, E. H. M. *et al.* Leflunomide–hydroxychloroquine combination therapy in patients with primary Sjögren’s syndrome (RepurpSS-I): A placebo-controlled, double-blinded, randomised clinical trial. *Lancet Rheumatol.* (2020). 10.1016/S2665-9913(20)30057-6 38273473

[22] Pareek A (2014). Efficacy and safety of hydroxychloroquine in the treatment of type 2 diabetes mellitus: A double blind, randomized comparison with pioglitazone. Curr. Med. Res. Opin..

[23] Schrezenmeier E, Dörner T (2020). Mechanisms of action of hydroxychloroquine and chloroquine: Implications for rheumatology. Nat. Rev. Rheumatol..

[24] Geleris J (2020). Observational study of hydroxychloroquine in hospitalized patients with Covid-19. N. Engl. J. Med..

[25] Magagnoli, J. *et al.* Outcomes of hydroxychloroquine usage in United States veterans hospitalized with Covid-19. *medrxiv* (2020). 10.1016/j.medj.2020.06.001 PMC7274588 32838355

[26] Chen, Z. *et al.* Efficacy of hydroxychloroquine in patients with COVID-19: Results of a randomized clinical trial. *MedRxiv* (2020).

[27] Tang, W. *et al.* Hydroxychloroquine in patients with COVID-19: An open-label, randomized, controlled trial. *MedRxiv* (2020).

[28] Moher D, Liberati A, Tetzlaff J, Altman DG (2009). Preferred reporting items for systematic reviews and meta-analyses: The PRISMA statement. Ann. Intern. Med..

[29] Abd-Elsalam S (2020). Hydroxychloroquine in the treatment of COVID-19: A multicenter randomized controlled study. Am. J. Trop. Med. Hyg..

[30] Cavalcanti AB (2020). Hydroxychloroquine with or without azithromycin in mild-to-moderate Covid-19. N. Engl. J. Med..

[31] Chen, C.-P. *et al.* A Multicenter, randomized, open-label, controlled trial to evaluate the efficacy and tolerability of hydroxychloroquine and a retrospective study in adult patients with mild to moderate Coronavirus disease 2019 (COVID-19). *medRxiv* (2020). 10.1371/journal.pone.0242763 PMC7710068 33264337

[32] Chen J (2020). A pilot study of hydroxychloroquine in treatment of patients with common coronavirus disease-19 (COVID-19). J. Zhejiang Univ..

[33] Chen, L. *et al.* Efficacy and safety of chloroquine or hydroxychloroquine in moderate type of COVID-19: A prospective open-label randomized controlled study. *medRxiv* (2020).

[34] Horby, P. *et al.* (medRxiv, 2020).

[35] Tang, W. *et al.* Hydroxychloroquine in patients with mainly mild to moderate coronavirus disease 2019: Open label, randomised controlled trial. *bmj* **369** (2020). 10.1136/bmj.m1849 PMC7221473 32409561

[36] Consortium, W. S. T. (2021). Repurposed antiviral drugs for COVID-19—interim WHO SOLIDARITY trial results. N. Engl. J. Med..

[37] Kamran, S. M. *et al.* Clearing the fog: Is hydroxychloroquine effective in reducing coronavirus disease-2019 progression? A randomized controlled trial. *Cureus* **13** (2021). 10.7759/cureus.14186 PMC8083993 33936897

[38] Magnus Nakrem, L. *et al.* A pragmatic randomized controlled trial reports the efficacy of hydroxychloroquine on coronavirus disease 2019 viral kinetics. *Res. Sq.* (2021). 10.1038/s41467-020-19056-6 PMC7576792 33082342

[39] Chacko, J., Brar, G. & Premkumar, R. Hydroxychloroquine in COVID-19: A systematic review and meta-analysis. *medRxiv* (2020).

[40] Sarma P (2020). Virological and clinical cure in COVID-19 patients treated with hydroxychloroquine: A systematic review and meta-analysis. J. Med. Virol..

[41] Shamshirian, A. *et al.* Hydroxychloroquine versus COVID-19: A periodic systematic review and meta-analysis. *MedRxiv* (2020).

[42] Pathak SK (2020). No benefit of hydroxychloroquine in COVID-19: Results of systematic review and meta-analysis of randomized controlled trials”. Diabetes Metab. Syndr..

[43] Gautret P (2020). Hydroxychloroquine and azithromycin as a treatment of COVID-19: Results of an open-label non-randomized clinical trial. Int. J. Antimicrob. Agents.

[44] Sandeep, S. & McGregor, K. Energetics Based Modeling of Hydroxychloroquine and Azithromycin Binding to the SARS-CoV-2 Spike (S) Protein-ACE2 Complex. (2020).

[45] Yao X (2020). In vitro antiviral activity and projection of optimized dosing design of hydroxychloroquine for the treatment of severe acute respiratory syndrome coronavirus 2 (SARS-CoV-2). Clin. Infect. Dis..

[46] Gueyffier F, Cucherat M (2019). The limitations of observation studies for decision making regarding drugs efficacy and safety. Therapies.

[47] Fiolet, T. *et al.* Effect of hydroxychloroquine with or without azithromycin on the mortality of COVID-19 patients: A systematic review and meta-analysis. *Clin. Microbiol. Infect.* (2020). 10.1016/j.cmi.2020.08.022 PMC7449662 32860962

[48] Arshad S (2020). Treatment with hydroxychloroquine, azithromycin, and combination in patients hospitalized with COVID-19. Int. J. Infect. Dis..

[49] Li H (2014). Meta-analysis of the adverse effects of long-term azithromycin use in patients with chronic lung diseases. Antimicrob. Agents Chemother..

[50] Tang C, Godfrey T, Stawell R, Nikpour M (2012). Hydroxychloroquine in lupus: Emerging evidence supporting multiple beneficial effects. Intern. Med. J..

[51] Arasiewicz, H., Samborska, M., Salwowska, N., Zbiciak-Nylec, M. & Brzezińska-Wcisło, L. Hydroxychloroquine–drug characterization and the most frequently observed adverse reactions in the group of patients with diagnosed alopecia cicatricans.

[52] Srinivasa A, Tosounidou S, Gordon C (2017). Increased incidence of gastrointestinal side effects in patients taking hydroxychloroquine: A brand-related issue?. J. Rheumatol..

[53] Pelle MT, Callen JP (2002). Adverse cutaneous reactions to hydroxychloroquine are more common in patients with dermatomyositis than in patients with cutaneous lupus erythematosus. Arch. Dermatol..

[54] Detert J (2014). Hydroxychloroquine in patients with inflammatory and erosive osteoarthritis of the hands (OA TREAT): Study protocol for a randomized controlled trial. Trials.

[55] Cavazzana I (2009). Treatment of lupus skin involvement with quinacrine and hydroxychloroquine. Lupus.

[56] Modi JV (2017). Dose response relationship of hydroxychloroquine sulphate in the treatment of rheumatoid arthritis: A randomised control study. Int. J. Pharm. Sci. Res..

[57] Yogasundaram H (2014). Hydroxychloroquine-induced cardiomyopathy: Case report, pathophysiology, diagnosis, and treatment. Can. J. Cardiol..

[58] Joyce E, Fabre A, Mahon N (2013). Hydroxychloroquine cardiotoxicity presenting as a rapidly evolving biventricular cardiomyopathy: Key diagnostic features and literature review. Eur. Heart J. Acute Cardiovasc. Care.

[59] Cairoli E (2015). Cumulative dose of hydroxychloroquine is associated with a decrease of resting heart rate in patients with systemic lupus erythematosus: A pilot study. Lupus.

[60] Pandya HK, Robinson M, Mandal N, Shah VA (2015). Hydroxychloroquine retinopathy: A review of imaging. Indian J. Ophthalmol..

[61] Weiner A, Sandberg MA, Gaudio AR, Kini MM, Berson EL (1991). Hydroxychloroquine retinopathy. Am. J. Ophthalmol..

[62] Iselin K, Marti P, Pless M (2016). Hydroxychloroquine-Induced Retinal Toxicity. Klin. Monbl. Augenheilkd..

[63] Marmor MF, Kellner U, Lai TY, Melles RB, Mieler WF (2016). Recommendations on screening for chloroquine and hydroxychloroquine retinopathy (2016 revision). Ophthalmology.

[64] Jorge A, Ung C, Young LH, Melles RB, Choi HK (2018). Hydroxychloroquine retinopathy—Implications of research advances for rheumatology care. Nat. Rev. Rheumatol..

